# Relation between Birth Weight, Growth, and Subclinical Atherosclerosis in Adulthood

**DOI:** 10.1155/2015/926912

**Published:** 2015-01-14

**Authors:** Maria Helena Valente, Filumena Maria da Silva Gomes, Isabela Judith Martins Benseñor, Alexandra Valéria Maria Brentani, Ana Maria de Ulhôa Escobar, Sandra J. F. E. Grisi

**Affiliations:** ^1^Department of Pediatrics, Medical School, University of São Paulo (USP), 01246-903 São Paulo, SP, Brazil; ^2^Department of Clinical Medicine, Medical School, University of São Paulo (USP), 01246-903 São Paulo, SP, Brazil

## Abstract

*Background and Objectives.* Adverse conditions in the prenatal environment and in the first years of life are independently associated with increased risk for cardiovascular disease. This paper aims to study the relation between birthweight, growth in the first year of life, and subclinical atherosclerosis in adults. *Methods.* 88 adults aged between 20 and 31 were submitted to sociodemographic qualities, anthropometric data, blood pressure measurements, metabolic profile, and evaluation of subclinical atherosclerosis. *Results.* Birthweight <2,500 grams (g) was negatively correlated with (a) increased waist-to-hip ratio (WHR), according to regression coefficient (RC) equal to −0.323, 95% CI [−0.571, −0.075] *P* < 0.05; (b) diastolic blood pressure (RC = −4.744, 95% CI [−9.017, −0.470] *P* < 0.05); (c) low HDL-cholesterol (RC = −0.272, 95% CI [−0.516, −0.029] *P* < 0.05); (d) frequency of intima-media thickness (IMT) of left carotid >75th percentile (RC = −0.242, 95% CI [−0.476, −0.008] *P* < 0.05). Birthweight >3,500 g was associated with (a) BMI >25.0 kg/m^2^, (RC = 0.317, 95% CI [0.782, 0.557] *P* < 0.05); (b) increased waist circumference (RC = 0.284, 95% CI [0.054, 0.513] *P* < 0.05); (c) elevated WHR (RC = 0.280, 95% CI [0.054, 0.505] *P* < 0.05); (d) minimum subcutaneous adipose tissue (SAT) (RC = 4.354, 95% CI [0.821, 7.888] *P* < 0.05); (e) maximum SAT (RC = 7.095, 95% CI [0.608, 13.583] *P* < 0.05); (f) right lobe of the liver side (RC = 6.896, 95% CI [1.946, 11.847] *P* < 0.001); (g) frequency's right lobe of the liver >75th percentile (RC = 0.361, 95% CI [0.169, 0.552] *P* < 0.001). Weight gain in the first year of life was inversely correlated with (a) mean IMT of left carotid (RC = −0.046, 95% CI [−0.086, −0.006] *P* < 0.05; (b) frequency IMT of left carotid >75th percentile (RC = −0.253, 95% CI [−0.487, −0.018] *P* < 0.05); (c) mean IMT (RC = −0.038, 95% CI [0.073, −0.002] *P* < 0.05); (d) the frequency of the mean IMT >75th percentile (RC = −0.241, 95% CI [−0.442, −0.041] *P* < 0.05). *Conclusions.* Adults birthweight <2,500 g and >3,500 g and with insufficient weight gain in the first year of life have showed different metabolic phenotypes, but all of them were related to subclinical atherosclerosis.

## 1. Introduction

Environmental factor that acts in the early life may influence the risk of diseases in adult life [1]. Barker and Osmond [2] first associated the adverse conditions at prenatal environment with the risk for cardiovascular disease in adults, with studies that showed that individuals with low birth weight or restricted intrauterine growth show increased risk for developing chronic cardiovascular disease [3–5]. Impaired growth during childhood is also considered important for the risk of future cardiovascular events in adulthood [6].

A series of worldwide epidemiological studies extended the initial observations on the association between pre- and postnatal growth and cardiovascular disease, in order to include relations between initial growth patterns and increased risk for hypertension, impaired glucose tolerance, type 2 diabetes, insulin resistance, and obesity in adult life [7–9].

Given the increasing prevalence of obesity in the world, a variant of the Barker hypothesis was created, in which excessive nutrition during pregnancy and high birth weight may be related to chronic disease in adult life, such as obesity and correlated conditions in adulthood [10].

Just as intrauterine growth restriction (IURG) forces the fetus to restructure the metabolic machinery, exposure to excessive maternal nutrition (secondary to the current epidemic of diabetes and obesity) may be associated with parallel increase of macrosomic births. Recent studies show strong associations between neonatal adiposity and metabolic disorders in adulthood [11].

Therefore, this paper aims to study the relation between birth weight, growth in the first year of life, and risk factors for cardiovascular disease and subclinical atherosclerosis in adults of the “Professor Samuel B. Pessoa” Health Center-School (CSE) of the Faculty of Medicine from the University of São Paulo.

## 2. Methods

The study had an analytical and observational circumscription. It was conducted in adults who had medical records in the CSE as newborns or infants from the period between 1977 and 1989 and who are still being followed in the Health Center-School nowadays. All subjects agreed to participate in the study and gave the Term of Free and Informed Consent Form (ICF) in writing, according to the Resolution from 1996 of the Brazilian National Health Council.

### 2.1. The Research Project

The research project was developed at the Health Center-School (CSE) from the Medical School of the University of São Paulo, where practices of primary health care are developed with adults selected from the existing medical records. Other health centers apart from the CSE collaborated for this study: Children's Institute from the “Hospital das Clínicas” of the University of São Paulo (ICr-HCFMUSP) and “Hospital Universitário” of the same university (HU-USP). The following inclusion criteria for participation in the study were considered: subjects must currently have been between 20 and 31 years old, which means, being born between 1977 and 1989 for both sexes; subjects must have read and signed the Term of Free and Informed Consent Form (ICF); their birth weight and perinatal data must have been recorded in their medical records; the subjects should have been followed at the CSE when they were at the pediatric age group; and they should also be currently subjected to regular medical monitoring in the CSE.

### 2.2. Individuals from the Research

**Figure 1:**
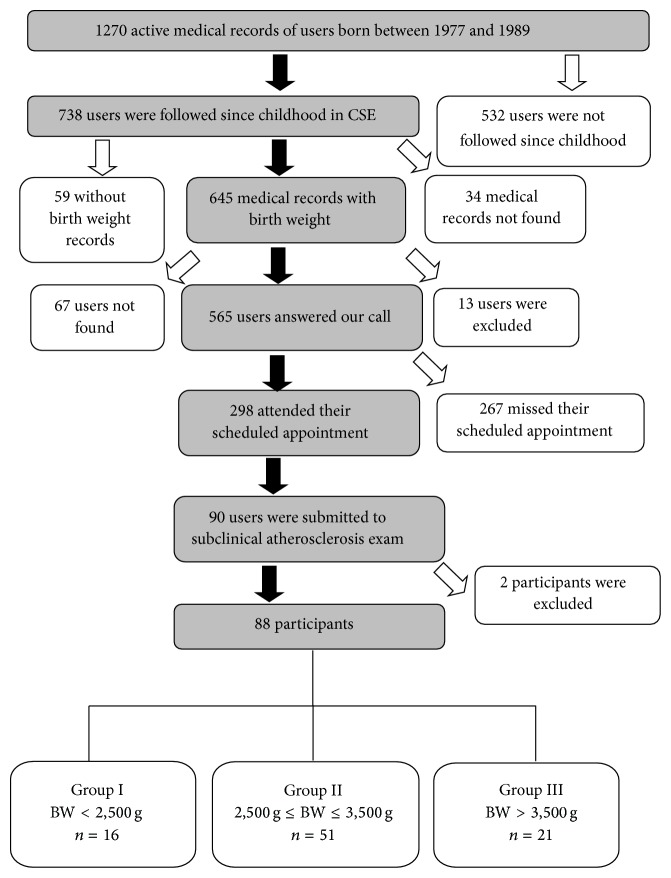
Flowchart of the study's group constitution considering the participants' birth weight.

In order to form the groups of study, we reviewed 738 medical records, of which 645 had neonatal data required in the inclusion criteria. Of these ones, 298 users answered our call and signed the Term of Free and Informed Consent Form. Once the study included the evaluation of atherosclerosis by ultrasound and by graphical methods, and considering that the frequency of cardiovascular disease mortality is 31.3% in our midst [12], 90 randomly chosen users were submitted to the examinations. At this moment, two individuals were then excluded, which means that 88 subjects were submitted to the evaluation of atherosclerosis. One participant did not attend the ultrasound examination and seven people missed their scheduled appointment for their pulse wave velocity (PWV) examinations and their electrocardiogram. Two individuals were not able to have their abdominal fat minimum and maximum measured because they were too emaciated. The flowchart of the establishment of study groups in relation to birth weight is summarized in Figure 1.

**Figure 2:**
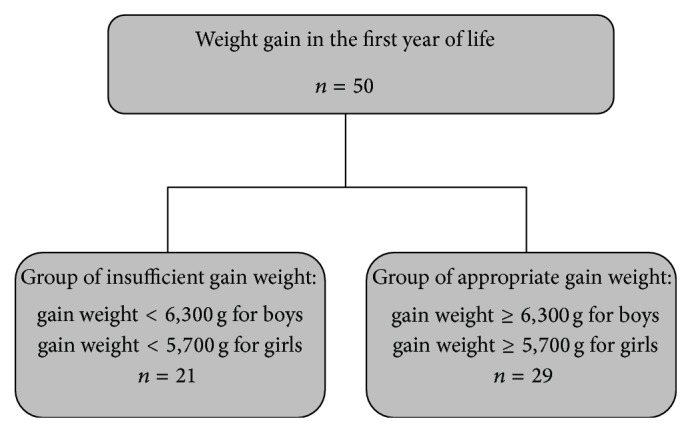
Flowchart of the constitution of the study groups based on weight gain in first year of life, according to gender.

The weight gain in the first year of life was obtained in the medical records of 50 subjects among the 88 participants. These medical records were divided in two groups (see Figure 2).

### 2.3. Constitution of the Research Groups

The 88 subjects were divided into groups according to two criteria: birth weight and weight gain in the first year of life. We used two values as references to form the groups: 2,500 grams and 3,500 grams. A birth weight below 2,500 grams is considered as low birth weight [13]. The choice of the value of 3,500 grams takes as reference the work of Kumar et al. [14], which considers this high birth weight as 97th percentile for term newborns. We assumed the values of 6,300 grams for male subjects and 5,700 for female subjects as cutoff points for weight gain in the first year of life. These values result from the difference between expected weight at the age of 1 and the 50th percentile birth weight, which is based on curves and tables from the World Health Organization [15]. The study groups were as follows.

- Birth weight:
  - *Group I*: birth weight lower than 2,500 grams.
  - *Group II*: birth weight between 2,500 grams and 3,500 grams.
  - *Group III*: birth weight greater than 3,500 grams.
- Weight gain in the first year of life:
  - *Group of insufficient weight gain*: weight gain < 6,300 grams for boys and < 5,700 grams for girls.
  - *Group of adequate weight gain*: weight gain ≥ 6,300 grams for boys and ≥ 5,700 grams for girls.

### 2.4. Clinical Parameters

The selected subjects had their data collected through a structured questionnaire which addressed sociodemographic characteristics (age, gender, ethnicity, educational level, and per capita income), presence of cardiovascular risk factors (smoking, alcohol consumption, and drug use), previous diagnoses (arterial hypertension, diabetes mellitus, dyslipidemia, and cardiovascular disease), conditions of birth (weight, gestation period), weight and height development in early life, and diseases in the neonatal period and childhood.

### 2.5. Anthropometric Parameters

Anthropometric parameters were measured using techniques and equipment in accordance with the recommendations of the World Health Organization (WHO) and the* Centers for Disease Control and Prevention* [16]. The following anthropometric measurements were registered: weight, height, waist and hip circumference, calculation of waist-hip ratio (WHR), and body mass index (BMI) [16]. For evaluation of centripetal obesity in waist circumference, the following values were considered normal: 102 cm or less in men and 88 cm or less in women [17]. Concerning WHR, it was considered normal value for subjects aged between 20 and 29 who showed a ratio equal to or less than 0.83 in females and less than or equal to 0.94 in males. For those aged between 30 and 39, their WHR was considered normal when it was equal to or less than 0.84 in female subjects and when it was equal to or less than 0.96 in male subjects [18]. The resting blood pressure and heart rate were measured three times in the sitting position after 5 minutes of rest, using the automatic sphygmomanometer, brand microlife BP 3BTO-A, with the clamp suitable for each individual. Hypertension was indicated when there was reference to a previous medical diagnosis of hypertension, or when there was use of medications for high blood pressure, or when systolic blood pressure (SBP) was ≥140, or when the diastolic blood pressure (DBP) was ≥90 mm Hg [19].

### 2.6. Laboratory Parameters

Participants were considered diabetic when they had had a previous history of diabetes mellitus diagnosis, when they had used medications for diabetes treatment, or when their fasting glucose level was greater than or equal to 126 mg/dL [20]. Participants were classified according to the guidelines of the* American Heart Association*, which considers the values of triglycerides that are equal to or greater than 150 mg/dL and the values of HDL cholesterol that are lower than 40 mg/dL to be altered. LDL-cholesterol values are considered altered depending on the risk rating of the subject [17, 21]. Participants were classified as having dyslipidemia when they had had a previous dyslipidemia diagnosis, when they had been previously submitted to treatment for dyslipidemia, or when they presented high triglycerides and/or low HDL cholesterol and/or high LDL-c [17, 21].

### 2.7. Cardiovascular Risk Profile

The established criteria for metabolic syndrome diagnosis consist in the presence of three or more of the following items [22]: increase of abdominal circumference greater than 88 cm in women and greater than 102 cm in men; increase of plasma triglycerides greater than or equal to 150 mg/dL; reduced levels of HDL cholesterol (below 40 mg/dL for men and less than 50 mg/dL for women); increased blood pressure levels with systolic blood pressure equal to or greater than 130 mm Hg and/or diastolic blood pressure equal to or greater than 85 mm Hg; and increased levels of fasting plasma glucose equal to or greater than 100 mg/dL.

### 2.8. Indirect Evaluation of Subclinical Atherosclerosis by Ultrasound—Measurement of Subcutaneous Adipose Tissue (SAT) and Hepatic Fat

Fat layers of the abdominal wall thickness (minimum, maximum, and peritoneal) and visceral fat (liver) were measured by ultrasound. The ultrasound images were obtained with* Toshiba Aplio XG* equipment with a 7.5 MHz linear transducer. The images of the liver were obtained with standard equipment (*Toshiba SSA-770A Aplio*, Japan) and broadband convex transducer (*PVT-375BT*) with center frequency of 3.5 MHz (2.5–5.5 MHz). The images were recorded and measured in workstation with the software* IMAGE TOM ARENA-TEC*.

### 2.9. Indirect Evaluation of Subclinical Atherosclerosis by Ultrasound—Measurement of Mean Carotid Intima-Media Thickness (IMT)

The standardizing of the ultrasound method for measurement of the thickness of the carotid intima-media followed the protocol of the Center for Clinical and Epidemiological Research of HU-USP. The bifurcation of the carotid arteries was analyzed in a length of 3 centimeters (cm) in search for plates. The image of the common carotid over 1 cm was analyzed as well, beginning 1 cm below the bifurcation of the common carotid. The intima-media thickness (IMT) was measured in all participants in a standardized way with equipment* Toshiba* (*Aplio XG*) with a 7.5 MHz linear transducer and was recorded from the sequence of scans in the transverse axis, with duration of 8 seconds. The satisfactory ultrasound images in B-mode of the carotids referred to the evaluation of the clear interfaces of the arterial walls in the longitudinal axis and in the arterial anatomical repairs, with the intima-lumen interfaces and media-adventitia displayed on the distal arterial wall, furthest from the transducer. The interpretation of the examination was centralized and automated using the software* MIA* with images of three cardiac cycles. Mean and maximum values of the IMT of the left and right carotids were calculated, as well as the mean between left and right carotid values. The measure of IMT was performed on the image of the posterior wall of the distal segment of the common carotid, through which measurements of vessel size, vascular lumen, and the distal wall's IMT during the cardiac cycles were obtained. The software measures' algorithm calculated the distance between the lines that delineated the anatomical structure of the vessels and calculated also the maximum and average IMT values, with standard deviation. In the evaluation of the intima-media thickness (IMT) of the carotid arteries the following parameters were considered: average IMT of the left carotid (LC's IMT) in millimeters; average IMT of the right carotid (RC's IMT) in millimeters; and the mean between left carotid's IMT and right carotid's IMT ((LC's IMT + RC'S IMT)/2) in millimeters.

### 2.10. Indirect Evaluation of Subclinical Atherosclerosis by Ultrasound—Measure of the Pulse Wave Velocity (PWV)

The carotid-femoral pulse wave velocity (PWV) was measured with a validated automatic device (*Complior, Artech Medicale*, France) with the participant in the dorsal decubitus position. Before the measurement of PWV, blood pressure was obtained in the lying position with oscillometric equipment (*Onrom* HRM CP 705) on the right arm. The distance from the sternal notch to the right femoral pulse was obtained using tape measure. The pulse sensors were positioned in the carotid arteries and in the right femoral pulse, allowing visualization of pulse waves on a computer screen. A software identified the pulse waves with good recording quality. The PWV was calculated by dividing the distance from the sternal notch to the femoral pulse by the time lag between the carotid and femoral pulses using the average of 10 consecutive cardiac cycles in regular heartbeat. All PWV examinations were registered and sent for analysis at the Center of Cardiovascular Physiology Reading in the Center for Clinical and Epidemiological Research of HU-USP.

### 2.11. Indirect Evaluation of Subclinical Atherosclerosis by Ultrasound—Evaluation of Alterations in the Electrocardiogram (ECG)

This examination was conducted using 12 derivations with records of the heart's electrical activity captured by electrodes placed on arms and legs of the subject. After the record, pace, cardiac frequency, and electrocardiogram (ECG) traces tests were made in the Laboratory of Electrocardiography of the Heart Institute (Instituto do Coração (INCOR)/HC-FMUSP).

### 2.12. Statistical Analysis

The database was created using the* Statistical Package for Social Science for Windows* (SPSS) software, version 19.0 (Chicago, Il, USA), and spreadsheets were created using* Microsoft Excel 2002*. In order to achieve the objectives of this paper, the descriptive statistical analysis of parametric variables was performed by calculating the mean, standard deviation and standard error with the results expressed through these parameters. The analysis of quantitative variables was performed by observing the minimum and maximum values and also by calculating means and standard deviations using analysis of variance (*Anova*) when it was necessary to compare one group to another. Absolute and relative frequencies were calculated for the analysis of* boolean* variables (presence or absence of a certain parameter). Pearson's Chi-squared test or Fisher's exact test was used in order to test the homogeneity of groups in relation to the proportions. In the cases in which the assumption of normality was rejected,* Mann-Whitney* nonparametric test was performed. In all analyses, a significance level of 5% was adopted. Multivariate regressions were performed using the* Stata 12 Statistical Software Package.*

## 3. Results

The analysis of the sociodemographic characteristics of the study group showed that it had a similar frequency of white and black people. The average age was 25.5 years, with a predominance of female subjects. Over half of the subjects (62.5%) reported that they had completed high school. 64.8% had an income per capita higher than 1 minimum wage. Smoking, alcohol consumption, and drug abuse were mentioned, respectively, by 23.9%, 10.2%, and 5.7% of the participants. Previous personal history of hypertension, dyslipidemia, and mental illness were mentioned, respectively, by 6.8%, 2.3%, and 8% of the subjects. These data are displayed in Table 1.

The anthropometric profile showed that the average body mass index (BMI) of the entire group was 24.6 kg/m^2^. A BMI > 25.0 kg/ m^2^ was found in 37.5% of the group while obesity (BMI > 30.0 kg/m^2^) in 10.2% of the participants. Increased waist circumference (WC) and increased waist-hip ratio (WHR) were detected in 29.5% and 46.6% of adults, respectively. The study of the blood pressure levels showed that 12.5% of the adults could be diagnosed with hypertension.

### 3.1. Characterization according to Birth Weight

The anthropometric profile and blood pressure measurements in relation to birth weight are described in Table 2.

Low birth weight (<2,500 grams) was inversely correlated with increased waist-to-hip ratio according to the regression coefficient (RC) −0.323, confidence interval (CI) 95% [−0.571, −0.075]  *P* < 0.05, and also with increased levels of diastolic blood pressure (RC = −4.744, 95% CI [−9.017, −0.470]  *P* < 0.05). Birth weight greater than 3,500 grams was associated with excessive body mass index (BMI ≥ 25 kg/m^2^) (RC = 2.832, 95% CI [0.433, 5.233]  *P* < 0.05), excessive weight or BMI ≥ 25 kg/m^2^ (RC = 0.318, 95% CI [0.782, 0.557] *P* < 0.05), increased high waist circumference (CR = 0.284, 95% CI [0.054, 0.513] *P* < 0.05), and increased waist-hip ratio (RC = 0.280, 95% CI [0.054, 0.505] *P* < 0.05), as it is shown in Table 2.

No participant had diabetes mellitus. The mean blood glucose, total cholesterol, LDL-cholesterol, HDL-cholesterol, and triglycerides among the study participants were, respectively, 81.7 (±6.5), 160.1 (±35.6), 90.0 (±23.9), 47.7 (±13.1), and 93 mg/dL (±78.1). Dyslipidemia was diagnosed in 29.5% of the subjects. Metabolic syndrome was found in 8.0% of them.

Low birth weight was inversely correlated with low HDL-cholesterol values, according to RC = −0.272, CI 95% [−0.516, −0.029], with *P* < 0.05. Birth weight greater than 3,500 grams was associated with fasting glucose levels (RC = 3.808, IC 95% [0.558, 7.058], *P* < 0.05), registered in Table 2.

The mean measurements of minimum, maximum, peritoneal, and visceral (of right lobe of the liver) subcutaneous abdominal fat (SAT) were, respectively, 11.3 (±7.1), 21.4 (±12.7), 13.2 (±5), and 96.0 (±11) mm. The frequencies of minimum, maximum, and peritoneal SAT above the 75th percentile were 24.7%, 24.7%, and 24.1%, respectively. Analysis of the visceral fat showed that 24.1% of the participants had right liver lobe above the 75th percentile and that 18.4% of the adults had abnormal liver echogenicity.

Birth weight greater than 3,500 grams was positively associated with mean values of minimal subcutaneous abdominal fat (SAT) (RC = 4.354, 95% CI [0.821, 7.888] *P* < 0.05), mean values of maximum SAT (RC = 7.095, CI 95% [0.608, 13.583] *P* < 0.05), increased right liver lobe size (RC = 6.896, 95% CI [1.946, 11.847] *P* < 0.01), and the frequency of right liver lobe above the 75th percentile (RC = 0.361, CI 95% [0.169, 0.552], *P* < 0.01). These data are shown in Table 2.

The mean of the left carotid's average and maximum IMT were 0.47 (±0.08) and 0.58 (±0.10) mm, respectively. The frequencies of the left carotid's average and maximum IMT above the 75th percentile were 24.1% for both measures. The mean of the right carotid's average and maximum IMT were 0.46 (±0.07) and 0.57 (±0.09) mm, respectively. The frequencies of the right carotid's average and maximum IMT above the 75th percentile were 23% and 21.8%, respectively. The mean of the mean IMT (LC + RC)/2 was 0.47 (±0.06) mm. The frequency of mean IMT (LC + RC)/2 above the 75th percentile was 18.4%.

Low birth weight was inversely correlated with left carotid's average IMT above the 75th percentile (RC = −0.242, CI 95% [−0.476, −0.008], *P* < 0.05). Birth weight greater than 3,500 g was not correlated with mean values of IMT, nor with frequencies above the 75th percentile in the left and right carotids, in accordance with what is shown in Table 2.

The study of arterial elasticity showed average pulse wave velocity (PWV) equal to 7.91 (±1.12) meters/second, with 24.7% of the study's participants with PWV values above the 75th percentile. Low birth weight and birth weight greater than 3,500 g were not correlated with arterial stiffness through the pulse wave velocity exam.

Changes in the electrocardiogram (that can be correlated with atherosclerosis, such as ventricular preexcitation, atrioventricular block, atrial ectopic rhythm, left atrial enlargement, early repolarization, and ventricular hypertrophy) were detected in 14.8% of adults that participated in our study, but they were not associated with low birth weight, nor with increased birth weight (>3,500 g).

### 3.2. Characterization according to the Weight Gain in the First Year of Life

Information about weight gain in the first year of life of the study participants was obtained from 50 of the 88 adults. Weight gain in the first year of life was considered insufficient when lower than 6,300 grams for males and lower than 5,700 grams for females. Weight gain in the first year of life was considered adequate when equal to or higher than 6,300 grams for males and equal to or higher than 5,700 for females.

The insufficient weight gain in the first year of life was not correlated with anthropometric parameters, blood pressure measurements, metabolic profile, measurement of abdominal fat, or visceral fat measurement. The study of the relation between measurement of intima-media thickness (IMT) of the carotid arteries and weight gain in the first year of life is summarized in Table 3.

Insufficient weight gain in the first year of life was negatively correlated with left carotid's average IMT values (CR = −0.046, 95% CI [−0.086, −0.006]  *P* < 0.05), with frequency of left carotid's average IMT above the 75th percentile (CR = −0.253, 95% CI [−0.487, −0.018]  *P* < 0.05), with mean IMT ([LC + RC]/2) values (CR = −0.038, 95% CI [−0.073, −0.002]  *P* < 0.05), and with frequency of mean IMT ([LC + RC]/2) above the 75th percentile (CR = −0.241, 95% CI [−0.442, −0.041] *P* < 0.05).

## 4. Discussion

The study of the relation between the anthropometric profile of the participants and their birth weight showed that only birth weight greater than 3,500 grams was positively associated with body mass index (BMI) and the frequency of overweight, in accordance with what literature discusses about developmental origins of obesity [10, 23]. The hypothesis about the developmental origins of health and disease proposed by Barker initially showed a relation between fetal malnutrition and/or low birth weight and epigenetic influences associated with the development of common chronic diseases such as hypertension, renal disease, cardiovascular disease (CVD), diabetes, and other metabolic abnormalities in adulthood [1–5]. Recent studies have shown a strong association between metabolic disorders of adults and neonatal adiposity, reinforcing the argument that increased birth weight may be associated with increased adiposity in adulthood [10, 23, 24]. A variant of the original Barker hypothesis was formulated given the increasing prevalence of obesity, in which excessive nutrition or excessive weight gain during pregnancy could be associated with obesity and related conditions in the child's adulthood [10, 23, 24]. According to this concept, the excess maternal body weight or excessive weight gain in pregnancy disrupts intrauterine environment during fetal development, producing permanent changes in the hypothalamus, pancreatic islet cells, adipose tissue, or other biological systems that regulate body weight [10, 23, 24]. In humans, excessive birth weight predicts body mass index (BMI) and adverse health outcomes later in life. A recent meta-analysis performed with 643,902 individuals shows that birth weight greater than 4,000 grams can lead to a two-times increased risk of excessive weight in the long term, regardless of geographical origin/ethnicity, gender, socioeconomic status, and parents' weight [10]. The study of prenatal programming of obesity shows that children of diabetic mother present more body fat since birth, with rapid fat gain after their seventh year of life.

In this study, low birth weight was inversely associated with increased WHR (wait-hip ratio). Yajnik et al. observed that individuals born with lower weights showed a higher proportion of body fat in adult life when compared to those who were born heavier [25]. The highest proportion of visceral fat on those born smaller was correlated to a prioritization of the preductal circulation and to a demand for fat by the central nervous system, which would eventually become a part of the other tissues [25].

Birth weight greater than 3,500 grams was also correlated to increased abdominal fat and increased waist-rip ratio (WHR), with literature showing that individuals born heavier might also weigh more in adult life, but with an adequate corporal hormonal composition and with an adequate body fat distribution [26].

Regarding blood pressure, low birth weight was inversely related to levels of diastolic arterial blood pressure, with medical literature showing the relation between fetal growth restriction and arterial hypertension [27]. People who were born small due to severe intrauterine deprivation and who remained small during childhood presented a reduced number of nephrons and a reduced renal function, which can result in arterial hypertension [27]. Birth weight besides being inverse and significantly associated with levels of arterial pressure in adult life might also be related to magnitude of pressures fluctuations over time. Fetal programming is responsible for altering both renal and vascular structures, as well as the mechanisms of arterial pressure regulation [27].

The study of the metabolic profile of the subjects showed that low birth weight was inversely correlated with reduced HDL-cholesterol frequency. The first studies on fetal origins of dyslipidemia showed that low birth weight was associated with atherogenic lipid profile in adult life [28]. However, subsequent studies have provided only a weak support for the hypothesis that low birth weight can predict adult lipid levels [29, 30]. The association between low birth weight and serum lipids levels in adults appears to be too small to have clinical significance, as Milosevic et al. concluded from their literature review [24]. The accumulation of adverse social circumstances throughout life appears to be relevant to dyslipidemia in adulthood, while the contributions of birth weight seem inconsistent when present, eclipsed by the strong contribution of aggregate exposures over the life course [31].

Birth weight greater than 3,500 grams was positively associated with average fasting glucose levels. Population studies showed many possibilities, both with those with low birth weight and with those with higher birth weight linked with glycemic alterations, with studies describing a U-shaped curve [32].

Nonalcoholic fatty liver disease (NAFLD) has been associated with metabolic disorders and subclinical atherosclerosis, often accompanied by abnormal liver function [33]. The increase in visceral fat is associated with insulin resistance, whereas subcutaneous fat is more strongly associated with circulating concentrations of leptin and with general obesity [33].

The evaluation of subcutaneous and hepatic fat by ultrasonography of this study's subjects showed that birth weight higher than 3,500 grams was associated with more presence of both subcutaneous abdominal fat and visceral fat. Subcutaneous fat seems to be more strongly related to total body fat [33], while visceral fat is more strongly related to body mass index, abdominal waist, and total abdominal fat, with an inverse association between weight at birth and abdominal adiposity or trunk adiposity in children and adults [34]. Studies suggest the hypothesis that undernutrition in uterus and the consequent low birth weight are associated with smaller global dimension of the body in childhood, but with the increase of the propensity to abdominal adiposity, suggesting a direct effect of the intrauterine environment on the subcutaneous abdominal fat deposit. This supports the hypothesis that adiposity is programmed within the uterus [33, 34]. However, other studies have showed a U-shaped relation between birth weight and abdominal adiposity [35]. Factors that affect intrauterine growth may favor the propensity for injury in adult liver [36], with birth weight inversely associated with visceral fat rather than subcutaneous abdominal fat, only after adjusting for adult body mass index (BMI) [36]. The dependence of this association on the adjustment for BMI can be interpreted as supporting the hypothesis that fast postnatal weight gain promotes the accumulation of visceral fat rather than only the low birth weight alone [36]. The association of birth weight greater than 3,500 grams with visceral adiposity might be linked to body mass index of the subjects of this paper.

Increased intima-media thickness (IMT) of the carotid arteries is considered an important marker for cardiovascular disease [37]. This study observed the inverse correlation between low birth weight and the increase of the intima-media thickness of the carotids, as it was shown by the literature [38, 39]. Insufficient weight gain in the first year of life was also inversely associated with the increase of the carotids' IMT. Studies show a strong relation between weight at birth and the values of IMT in the later phases of life [38, 39]. Low birth weight, and specially the restraint of intrauterine growth, is pointed out as independent risk factor for atherosclerosis, measured by IMT, which seems to relate with the restraint of intrauterine growth over the structure of the artery, determining endothelial dysfunction and interference in the intensity of the inflammatory processes associated with atherosclerosis [38–40].

The mechanisms that explain the low birth weight, insufficient or excessive weight gain in the first year of life, and risk factors for cardiovascular disease in adulthood take into account the theory of Thrifty Phenotype [5–8] but are not yet fully known [41, 42]. The insufficient weight gain in the first year of life and the high rate of weight gain in the first year of life are linked to cardiovascular disease independent of birth weight [41, 42].

Despite the fact that the medical literature associated low birth weight with greater vascular rigidity through pulse wave velocity [43], in this study there was no evidence of this relation.

## 5. Conclusions

The present study confirms the hypothesis of the association between birth weight and risk for atherosclerotic vascular disease. This paper highlights that birth weight over 3,500 grams and low birth weight are two different phenotypic expressions, but they are both related to increased risk for cardiovascular disease and subclinical atherosclerosis. It also shows that insufficient weight gain in the first year of life is also associated with a greater risk for subclinical atherosclerosis disease.

## Figures and Tables

**Table 1:** Sociodemographic aspects and previous conditions of the subjects.

Characterization	
Age (years)	25.5 years (2.9)
Gender	
Female	52 (59.1%)
Ethnicity	
Afro-descendant	43 (48.9%)
Education (years)	
Has finished High school	55 (62.5%)
Per capita income	
≦One minimum wage	57 (64.8%)
Habits	
Smoking	21 (23.9%)
Alcohol consumption	9 (10.2%)
Drug use	5 (5.7%)
Previous medical conditions	
Arterial hypertension	6 (6.8%)
Diabetes mellitus	0 (0.0)
Dyslipidemia	2 (2.3%)
Cardiovascular disease	0 (0.0)
Mental disorder	7 (8.0%)

**Table 2:** Birth weight and subclinical atherosclerosis.

Regression coefficient (RC)/confidence interval (95%)			
	Low weight BW^7^ < 2.500 g	Increased weight BW > 3.500 g	Reference weight BW 2.500–3.500 g
	*n* = 16	*n* = 21	*n* = 51

^ 1^BMI (Kg/m^2^)	−1.518 (−4.159–1.122)	2.832^**^ (0.433–5.232)	20.811 (11.974–29.648)
^ 1^BMI > 25.0 Kg/m^2^	−0.065 (−0.329–0.198)	0.317^**^ (0.782–0.557)	−0.179 (−1.061–0.702)
Increased WC^2^ (%)	−0.046 (−0.299–0.206)	0.284^**^ (0.054–0.513)	0.715 (−0.129–1.560)
Increased WHR^3^ (%)	−0.323^**^ [−0.571–(−0.075)]	0.280^**^ (0.054–0.505)	0.495 (−0.333–1.325)
^ 4^DBP (mm Hg)	−4.744^**^ [−9.017–(−0.470)]	0.719 (−3.162–4.601)	63.885 (49.588–78.182)
Glucose level (mg/dL)	−0.400 (−3.977–3.176)	3.808^**^ (0.558–7.058)	76.058 (64.091–88.026)
HDL cholesterol < 40 mg/dL (%)	−0.272^**^ [−0.516–(−0.029)]	0.024 (−0.197–0.245)	0.203 (−0.612–1.019)

	*n* = 15	*n* = 21	*n* = 49

^ 5^Minimum SAT (mean and standard deviation)	−1.614 (−5.602–2.372)	4.354^**^ (0.821–7.888)	8.112 (−5.029–21.253)
Maximum SAT (mean and standard deviation)	−2.486 (−9.807–4.835)	7.095^**^ (0.608–13.583)	11.367 (−12.761–36.496)

	*n* = 16	*n* = 21	*n* = 50

Right lobe (mean and standard deviation)	−3.863 (−9.312–1.586)	6.896^***^ (1.946–11.847)	82.206 (64.017–100.395)
Right lobe > 75th percentile	−0.124 (−0.335–0.086)	0.361^***^ (0.169–0.552)	−0.366 (−1.070–0.337)

	*n* = 16	*n* = 21	*n* = 50

Mean IMT^6^ > 75th percentile	−0.242^**^ [−0.476–(−0.008)]	−0.039 (−0.251–0.172)	0.371 (−0.409–1.151)

^1^BMI: Body mass index.

^2^WC: Waist circumference.

^3^WHR: Waist-rip ratio.

^4^DBP: Diastolic blood pressure.

^5^SAT: Subcutaneous adipose tissue.

^6^IMT: Intima-media thickness.

^7^BW: Birth weight.

^**^ *P* < 0.05.

^***^ *P* < 0.001.

**Table 3:** Weight gain in the first year of life and atherosclerosis.

	Regression coefficient (RC)/confidence interval (95%)	
	Insufficient weight gain in the first year of life	Reference: adequate weight gain in the first year of life
	*n* = 50	
Left carotid	*n* = 21	*n* = 29
Average IMT^1^ (mean and standard deviation)	−0.046^**^ [−0.086–(−0.006)]	−0.357 (0.185–0.529)
Average IMT^1^ above the 75th percentile	−0.253^**^ [−0.487–(−0.018)]	0.421 (−0.579–1.422)
Average IMT^1^ (LC + RC)/2	*n* = 21	*n* = 29
Average IMT^1^ (LC^2^ + RC^3^)/2	−0.038^**^ [−0.073–(−0.002)]	0.375 (0.222–0.528)
Average IMT^1^ (LC + RC)/2 >75th percentile	−0.241^**^ [−0.442–(−0.041)]	−0.147 (−1.002–0.707)

^1^IMT: Intima-media thickness.

^2^Left carotid.

^3^Right carotid.

^**^ *P* < 0.05.
